# Anandamide-Modulated Changes in Metabolism, Glycosylation Profile and Migration of Metastatic Melanoma Cells

**DOI:** 10.3390/cancers14061419

**Published:** 2022-03-10

**Authors:** Anna Sobiepanek, Małgorzata Milner-Krawczyk, Paulina Musolf, Tomasz Starecki, Tomasz Kobiela

**Affiliations:** 1 Laboratory of Biomolecular Interactions Studies, Chair of Drug and Cosmetics Biotechnology, Faculty of Chemistry, Warsaw University of Technology, Noakowskiego 3, 00-662 Warsaw, Poland; mmilnerkrawczyk@ch.pw.edu.pl (M.M.-K.); paulina.musolf.stud@pw.edu.pl (P.M.); 2 Institute of Electronic Systems, Warsaw University of Technology, Nowowiejska 15/19, 00-665 Warsaw, Poland; tomasz.starecki@pw.edu.pl

**Keywords:** melanoma metastasis, anandamide, metabolism, glycosylation profile, lectin–glycan interaction, biophysical methods, migration

## Abstract

**Simple Summary:**

Anandamide (AEA) belongs to the group of endocannabinoids and possesses various regulatory properties in physiological as well as pathological processes occurring in the organism. In this research some basic biological tests were applied to investigate AEA-induced changes in cell metabolism and motility, as well as advanced biophysical methods for the determination of the differences in the cell glycosylation profile on a highly dangerous model of melanoma skin cancer, for which an effective therapy is not yet available. Our research suggests that anandamide treatment of metastatic melanoma cells increases the cell metabolism which leads to the reduction in the metastatic potential of cells in terms of the cell glycosylation profile and cell migration. In the view of our research, it can be presumed that anandamide usage in the combined therapy of advanced melanoma would be an advantage for the patient.

**Abstract:**

An effective therapy for advanced melanoma, a skin cancer with the highest mortality, has not yet been developed. The endocannabinoid system is considered to be an attractive target for cancer treatment. The use of endocannabinoids, such as anandamide (AEA), is considered to be much greater than as a palliative agent. Thus, we checked its influence on various signaling pathways in melanoma cells. Our investigation was performed on four commercial cell lines derived from different progression stages (radial WM35 and vertical WM115 growth phases, lymph node WM266-4 metastasis, solid tumor A375-P metastasis). Cell viability, glucose uptake, quantification of reactive oxygen species production, expression of selected genes encoding glycosyltransferases, quantification of glycoproteins production and changes in the glycosylation profile and migration, as well as in cell elastic properties were analyzed. The cell glycosylation profile was investigated using the biophysical profiling method—the quartz crystal microbalance with dissipation monitoring (QCM-D). Anandamide treatment of only metastatic cells resulted in: an increase in the cell metabolism, a decrease in *GFAT-1* and *DPM1* expression, followed by a decrease in L1-CAM glycoprotein production, which further influenced the reduction in the cell glycosylation profile and migration. Considering our results, AEA usage is highly recommended in the combined therapy of advanced melanoma.

## 1. Introduction

Most of the diagnosed melanoma skin cancers are developed from melanocytes due to the ultraviolet (UV) radiation-related induction of genetic mutations, immunosuppression and photoaging [1]. Although melanocytes produce the pigment melanin to protect the skin against the UV radiation, continuous exposure to this factor activates oncogenes and inactivates suppressor genes, disrupting specific signal transduction pathways regulating the cell cycle, cell differentiation and apoptosis [2]. Mostly, melanoma cells retain the ability to produce melanin and form brown or black pigmented lesions, but in rare cases amelanotic melanoma may occur when melanin synthesis is disrupted [3,4]. Melanoma detected in the early phase (mainly in the radial growth phase, RGP) is entirely curable thanks to tissue excision [5]. The vertical growth phase (VGP) is considered the most crucial moment in the process of melanoma development. These cells gain the ability not only to survive in the dermis but also to proliferate there. If these cells acquire a further ability to metastasize, the patient has minimal chances for long-term survival (more than a few months). This is because melanoma cells can spread through the lymph nodes and vessels; thus, it is difficult for the tumor cells to be eliminated from the whole organism [6,7,8]. That is why treatment allowing the slowdown of the migration or blocking the cells in the primary growth phases could help the patient before surgery can be performed.

Taking into consideration that the cases of melanoma incidence constitute only about approximately 2% of all cancers regardless of gender (for comparison, non-melanoma skin cancers, approximately 5–7% of cases depending on gender), but at the same time the statistics show that every 5th patient with melanoma will die (21% of patients suffering from melanoma) and every 16th patient with non-melanoma skin cancer (6% of patients suffering from these cancers) [9], the problem is serious and increases with each year. Early diagnosis may be an important solution to this problem, especially because an effective therapy for advanced melanoma is still under intensive research [10]. Radiotherapy has been proven to be useful in melanoma treatment, although melanoma cells can display an intrinsic radioresistance [11]. In general, it is applied mainly as an adjuvant therapy, for example, in postsurgical treatment, in cases where surgical treatment is associated with a high risk (head, neck, advanced age), and in palliative treatment (mainly the brain and bone metastases) [12]. Novel approaches in cancer treatment are focused on the better understanding of the cancer progression processes. Since cancers affect the immune system, immunotherapy including vaccines (synthetic ones consist of two elements: antigen and adjuvant), monoclonal antibodies (for the immune checkpoints such as cytotoxic T-lymphocyte-associated antigen-4, CTLA-4; or programmed death-1 protein, PD-1) or adoptive T-cells (a flexible and personalized therapy) can be applied (more details are available in [13,14,15]). On the other hand, chemotherapy is applied when surgical treatment or modern approaches (e.g., immune and targeted therapy) cannot be performed either for medical or economic reasons. Three groups of chemotherapy can be distinguished: single-agent chemotherapy, combined chemotherapy (polychemotherapy) and biochemotherapy (which is a combination of cytokines and cytostatic agents). The response rate for a single-agent therapy varies between 5% and 20%, with the overall survival between 5 and 11 months [16,17]. Many side effects occur during chemotherapy, including vomiting, nausea, alopecia, fatigue and bone marrow suppression. The response rates rise for polychemotherapy and biochemotherapy in comparison with single-agent therapy, but there is no significant change in the overall survival. Moreover, in the case of the combined therapies, an increased cytotoxicity and more adverse effects are observed, which decreases the quality of the patients’ lives. That is why chemotherapy is considered more as a palliative treatment than a curative one [17,18]. The action of chemotherapeutic agents is based mainly on the induction of apoptosis. Two groups based on the action mode can be distinguished: the phase-specific agents and phase-nonspecific agents. The first group relies on the fact that cells are most vulnerable during the S (synthesis of DNA) and M (mitotic) phases of the cell cycle. The DNA intercalating agents (anthracyclines) and antimetabolites (gemcitabine) inhibit the DNA synthesis in the S phase, whereas vinca alkaloids (vincristine, vinblastine) and taxanes (taxol, docetaxel) are the mitosis inhibitors. Therapeutics from the second group (platinum derivatives, alkylating agents) act during the entire cell cycle [19]. Unfortunately, resistance to apoptosis is often observed both pre-existing and acquired. Antiapoptotic action could be performed via the upregulation of antiapoptotic pathways or by the mutations inactivating proapoptotic genes [16,20]. The alkylating agent dacarbazine (DTIC), until 2011, was believed to be a standard treatment for patients with inoperable metastatic melanoma. Although it showed a tolerable side effect profile, a clinically significant impact on the overall survival was not observed. Similar efficacy and toxicity was registered for another alkylating agent, temozolomide. Moreover, the application of alkylating agents such as DTIC in the combined therapy did not show any survival benefits for patients in comparison with monochemiotherapy [20,21,22].

Discovering the molecular changes, which occur during the development of the disease, enables the application of the targeted therapy. Some of the well-known markers for cutaneous melanoma are B-raf, N-ras, c-Kit, *CDKN*2*A*, VDR, MC1R, MITF, TYR/TRP1/TRP2, HAPLN1 and CTLA4/PD-1/PD-L1; and for the uveal melanoma: GNAQ/GNA11, BAP1, SF3B1 and EIF1AX [23]. The problem in many therapies is their lack of specificity. Various mutations induce tumor development and cutaneous melanoma has one of the highest mutation frequencies among other tumors. The first agents acting on the MAPK pathway are the inhibitors of RAS farnesyl transferase (including Tipifarnib and Lonafarnib), which block the post-translational modification of RAS protein and in this way prevent the translocation of the protein into the cell membrane. The *BRAF* mutation, the most common in melanoma patients (over 50% of cases), also influences the activity of the MAPK pathway, resulting in the promotion of tumor proliferation and resistance to apoptosis. The most common BRAF kinase-specific inhibitors are Sorafenib (BAY 43-9006) and Vemurafenib (PLX4032). In single drug treatment, clinical trials with these inhibitors showed that response rates of 48% and 84% were obtained, but in later phases, most patients developed resistance to these drugs which led to melanoma progression or recurrence. However, encouraging effects were noted as a result of the combination of these inhibitors with other drugs, such as the so-called effectors of metabolism (e.g., inhibitors of metabolic enzymes or inhibitors of the electron transport chain complexes) [20,21,24,25].

To overcome this issue, the recommended therapeutic approach is the simultaneous use of substance combinations directed at various cell signaling pathways [21]. Due to the dual role in tumorigenesis, as well as in the inhibition of tumor growth and metastatic spread, the endocannabinoid system (ECS) is considered to be an attractive target for cancer treatment [26]. ECS is a widespread neuromodulatory system, which consists of three elements: cannabinoid receptors (CBs), endogenous cannabinoids (endocannabinoids) and enzymes responsible for the synthesis, transport and degradation of endocannabinoids. The homeostasis of many cellular processes such as proliferation, apoptosis, inflammatory and allergic processes, as well as embryological development depend on the proper function of the endocannabinoid system. The current research is focused on the involvement of ECS in cancer development and possible options for the cancer-regressive effect or anticarcinogenic potential of cannabinoids [27,28,29]. Although identified in 1992, anandamide (AEA) is still one of the best-known endogenous cannabinoids. It belongs to the large family of N-acylethanolamines (NAEs) and is the agonist ligand for both types of endocannabinoid receptors, but with a higher affinity for the CB1 receptor than CB2. AEA is produced in the body in small amounts in various pathways with the use of appropriate enzymes (a detailed description of the four most important biosynthesis pathways can be found in [30]). It was proven that AEA is uptaken by the selective protein carrier into the cells where its degradation (mainly by hydrolysis) is performed. This process can be catalyzed by one of the two enzymes: the integral protein of the intracellular membrane (fatty acid amide hydrolase, FAAH) or the protein present in lysosomes and Golgi apparatus (N-acylethanolamine acid amidase, NAAA) [31,32]. On the other hand, AEA can also be oxidized, which causes a decreased level of this endocannabinoid in the organism. The oxidized products of endocannabinoid metabolism could promote cancer development. For example, cyclooxygenase-2 (COX-2) catalyzes the formation of prostaglandin ethanolamides (PGEA), which does not bind to the CB receptors. However, these products are ligands for the NF-κB and PPAR receptors, which are involved mainly in the pro-inflammatory processes and lipid turnover. Lipoxygenase-2 (LOX-2) action leads to the formation of hydroxyeicosatetraenoic acid (HETE) derivatives, which are involved in the regulation of the organism’s inflammatory response [31].

The use of endocannabinoids as palliative agents in cancer therapy has been known for a long time. They are effective at suppressing pain, stimulating appetite and reducing chemotherapy-induced nausea and vomiting. Furthermore, the influence of AEA on various tumors has already been investigated in terms of the induction of apoptosis in melanoma cells [33], disabled production of several important growth factors by breast cancer cells (such as vascular endothelial growth factor, VEGF; [34]), and the inhibition of the epithelial-mesenchymal transition (EMT) of breast cancer cells [35]. Therefore, the main focus of our research was on the impact of AEA on the changes in metabolism, glycosylation profile and migration of melanoma cells from different progression stages. Due to our newly established biophysical methodology for melanoma theranostic purpose, using the quartz crystal microbalance with dissipation monitoring (QCM-D) and the direct lectin–glycan interaction approach [36], we were able to detect changes in the glycosylation profile of AEA-treated melanoma cells in real-time and right on the surface of the cells. As a model for this basic research, commercial melanoma cells derived from different progression stages (radial WM35 and vertical WM115 growth phases, lymph node WM266-4 metastasis, solid tumor A375-P metastasis) were utilized. These results together with the results received by means of the typical biological assays (viability assay, glucose uptake assay, quantification of reactive oxygen species assay, migration assay), biomolecular assays (such as reverse transcription-quantitative polymerase chain reaction, ELISA test, fluorescent staining) and biophysical investigation (atomic force microscopy in the force spectroscopy mode) enabled a detailed analysis of anandamide influence on melanoma cells to be performed.

## 2. Materials and Methods

### 2.1. Cell Lines

For these studies, several human melanoma cell lines from ATCC were used: primary radial growth phase (WM35, CRL-2807), vertical growth phase (WM115, CRL-1675), metastasis to the lymph node (WM266-4, CRL-1676) and solid tumor amelanotic metastasis (A375-P, CRL-3224). All melanoma cell lines were cultured in a RPMI-1640 medium (VWR) containing L-glutamine and supplemented with a 10% fetal bovine serum (FBS, Life Technologies, Waltham, MA, USA) as well as a 1% of a penicillin–streptomycin mixture (Life Technologies).

Cells were passaged with the use of a 0.05% trypsin/EDTA solution (Sigma Aldrich, Burlington, MA, USA). For the following experiments cells were seeded in appropriate amounts onto multi-well plates (details described in each method separately) and were cultured at 37 °C in an atmosphere of 95% air/5% CO_2_.

### 2.2. Anandamide Treatment

Anandamide (AEA, Tocris Bioscience, Bristol, UK) was diluted in DMSO (Avantor Performance Materials, Gliwice, Poland) and next several micromolar concentrations (0.05–10 µM) were prepared in an RPMI-1640 complete medium to evaluate the influence of AEA on melanoma cells. As control samples (CTR) DMSO at a final concentration of 0.07% *v*/*v* was used, which had no influence on cell properties. A total of 24 h after the cells were seeded, the old medium was replaced by a new complete medium with an appropriate AEA concentration and incubation was carried out for another 24 h at 37 °C.

### 2.3. MTT Assay

The difference in the mitochondrial dehydrogenase activity of living cells was studied by the MTT assay as described previously [37]. Cells were seeded in a 96-well plate at the density of 1 × 10^4^ cells/well. Next, they were treated with various concentrations of AEA for 24 h and the MTT assay was performed.

A 0.5 mg/mL solution of MTT salt (Sigma Aldrich) was prepared in an RPMI-1640 medium without phenol red (Sigma Aldrich) directly before adding it to the cells. The cells were washed with a PBS buffer (Lonza Group, Basel, Switzerland), the MTT working solution was added and incubation was carried out for 1 h at 37 °C. For the formazan release, the medium was removed and 100 μL of DMSO was added to each well. The absorbance was measured at 570 nm on a plate reader (Synergy™ H4 Hybrid Multi-Mode Microplate Reader, BioTek Instruments, Winooski, VT, USA). The results were expressed as a percent of cells treated with 0.07% *v*/*v* DMSO (CTR).

### 2.4. Crystal Violet (CV) Assay

The influence of AEA on melanoma cells was studied by the crystal violet assay, where CV binds to the negatively charged cell elements. That is why this assay shows the general cell number in the sample. Cells were seeded in 96-well plate at the density of 1 × 10^4^ cells/well and after treatment, the CV assay was performed.

Cells were washed with a PBS buffer and fixed with 3.7% paraformaldehyde (PFA, Sigma Aldrich) in PBS for 30 min at room temperature (RT). Cells were washed with PBS and stained with a CV/MeOH solution (0.05% CV salt (Sigma Aldrich) in 1% methanol (Avantor Performance Materials)) for 30 min at RT. Then, cells were washed three times with Milli-Q water. A releasing agent for CV (100% methanol) was added and plates were incubated for 5 min at RT. Absorbance was measured at 540 nm on a plate reader and the results were expressed as a percent of cells treated with 0.07% *v*/*v* DMSO (CTR).

### 2.5. FDA/PI Assay

In living cells, fluorescein diacetate (FDA, Sigma Aldrich) is hydrolyzed to the fluorescent fluorescein thanks to the esterase activity, whereas propidium iodine (PI, Sigma Aldrich) intercalates to DNA/RNA in cells with damaged membranes. Thus, this double staining allows living and dead cells in the samples to be registered. Cells were seeded in a 96-well plate at the density of 1 × 10^4^ cells/well. After treatment, the FDA/PI test was conducted.

A staining solution of 8 μg/mL FDA and 3 μg/mL PI was prepared in an RPMI-1640 medium without phenol red. Cells were washed with PBS and then incubated with a fresh FDA/PI working solution for 5 min at 37 °C. Next, cells were washed with PBS and a fresh portion of a PBS buffer was added to perform the fluorescent measurement on a plate reader at the following wavelengths 490_EX_/526_EM_ nm for FDA and 535_EX_/635_EM_ nm for PI. The results were expressed as a percent of cells treated with 0.07% *v*/*v* DMSO (CTR).

### 2.6. Glucose Uptake Assay

To study changes in cell metabolism connected with the uptake of glucose by melanoma cells a commercially available Glucose Uptake-Glo^TM^ assay (Promega Corporation, Madison, Wisconsin, USA) was performed according to the manufacturer’s recommendations. Cells were seeded in a 96-well plate at the density of 1 × 10^4^ cells/well and after treatment, the assay was performed.

Cells once washed with PBS were then incubated with 1 mM 2-deoxyglucose for 10 min at RT. Next, the STOP buffer and neutralization buffer were added to each well, and the plate was mixed well on a platform shaker. The received solutions were afterwards transferred to a new 96-well white plate, the 2DG6P detection reagent was added and the luminescence was read after 60 min incubation at RT. The results were first normalized by the number of living cells received from the FDA/PI test, and later expressed as a percent of cells treated with 0.07% *v*/*v* DMSO (CTR).

### 2.7. Reactive Oxygen Species (ROS) Quantification Test

The production of reactive oxygen species in melanoma cells was measured fluorescently after cell incubation with 2,7-dichlorodihydrofluorescein diacetate (DCF-DA, Sigma-Aldrich). Cells were seeded in a 96-well plate at the density of 2 × 10^4^ cells/well and after treatment, staining was performed.

A working dilution of 10 μM DCF-DA concentration in an RPMI-1640 medium without phenol red was prepared. Cells were washed with PBS and incubated with a DCF-DA solution for 30 min at 37 °C. Next, the solution was removed and the cells were washed with PBS. Then, the RPMI-1640 medium without phenol red was added to each well. At this time point, two additional controls were also prepared and added to the selected wells with cells: a positive control with 100 μM *tert*-butyl hydroperoxide (*t*BHP, Sigma Aldrich) and a negative control with 5 mM glutathione (GSH, Sigma Aldrich), both in medium without phenol red. The fluorescence read was conducted after 1 h incubation time with the new medium on a plate reader with the following wavelengths 485_EX_/525_EM_ nm. The results were first normalized by the number of living cells received from the FDA/PI test, and later expressed as a percent of cells treated with 0.07% *v*/*v* DMSO (CTR).

### 2.8. RNA Isolation Followed by the Reverse Transcription-Quantitative Polymerase Chain Reaction (RT-qPCR)

RT-qPCR analysis was used to determine the changes in the expression of selected glycosyltransferases in melanoma cells, which were collected by trypsinization in the amount of 1 × 10^6^ and the total RNA was extracted using TRI Reagent (Sigma Aldrich) according to the manufacturer’s instructions. Next, 1 µg of RNA was reverse transcribed into cDNA using the RevertAid First Strand cDNA Synthesis Kit (Thermo Fisher Scientific, Waltham, MA, USA) according to the producer’s instruction and qPCR was performed using the Power SYBR™ Green PCR Master Mix (Thermo Fisher Scientific) on the LightCycler 96 (Hoffmann-La Roch AG, Basel, Switzerland). The following sequences of primers were used: target gene *GFAT-1*-Fw 5′-AACTACCATGTTCCTCGAACGA-3′, Rv 5′-CTCCATCAAATCCCACACCAG-3′; target gene *DPM1*-Fw 5′-CTTCTCCGAGAGTGGAATCAAC-3′, Rv 5′-GCAGTTCCTAGTCCCAACTTTTT-3′ and reference gene *β-actin* Fw 5′-AATGTGGCCGAGGACTTTGAT-3′, Rv 5′-AGGATGGCAAGGGACTTCCTG-3′. The PCR cycling conditions were: 40 cycles of 15 s at 95 °C, 35 s at 59 °C and 35 s at 72 °C and the post-PCR melting curve was obtained using settings: 15 s at 95 °C, 1 min at 60 °C and 15 s at 95 °C. The relative fold gene expression was calculated using the 2^−∆Ct^ method [38] and the results were expressed as a percent of cells treated with 0.07% *v*/*v* DMSO (CTR).

### 2.9. ELISA In Situ

The in situ ELISA assay was used to quantify the amount of L1-CAM glycoprotein produced by melanoma cells. Cells were seeded in the 96-well plate at the density of 1 × 10^4^ per well and after treatment, the assay was conducted.

Cells were rinsed with PBS, fixed with a 3.7% PFA solution and permeabilized for 10 min with a 0.1% Triton X-100 (Sigma Aldrich) solution in PBS. Next, the unspecific binding sites on the cells were blocked for 30 min at RT with a standard blocking buffer—3% bovine serum albumin (BSA, Sigma Aldrich), 0.1% Tween 20 (Sigma Aldrich), 0.1% Triton X-100 in PBS. The overnight incubation with a mouse anti-L1-CAM antibody (1:500 = 0.4 µg/mL, R&D Systems, Minneapolis, MN, USA) in a PBST buffer (PBS with 0.025% Tween 20) as well as a mouse anti-actin antibody (1:200 = 7.5 µg/mL, Sigma Aldrich) in a 0.2% BSA/PBST buffer was carried out at 4 ℃. In the following stage, the goat anti-mouse IgG (whole molecule) antibody conjugated with horseradish peroxidase (HRP) (1:10,000, Sigma Aldrich) in PBST was added for 2 h in RT. A 1:1 mixture of 30% hydrogen peroxide (H_2_O_2_, Avantor Performance Materials) with 3,3′,5,5′-Tetramethyl benzidine (TMB) Liquid Substrate (Sigma Aldrich) was added and incubation was carried for 30 min at RT. A 2 M sulfuric acid (ready-to-use stop solution, Sigma Aldrich) was added to each well before absorbance was measured at 450 nm. The obtained results for the amount of L1-CAM protein was first normalized by the corresponding result of the actin amount for each sample and later expressed as a percent of cells treated with 0.07% *v*/*v* DMSO (CTR).

### 2.10. QCM-D Lectin–Glycan Measurements

A quartz crystal microbalance with dissipation monitoring (QCM-D, Q-Sense E1, Biolin Scientific AB, Vastra Frolunda, Sweden) was used for the lectin–glycan measurements of changes in the glycosylation profile of melanoma cells. Cells were seeded on the gold sensors coated with polystyrene (QSX 305, Biolin Scientific) placed in a 24-well plate at the density of 5 × 10^4^ cells/well. After a 24 h treatment with AEA, cells were fixed with a 3.7% PFA solution. All measurements were carried out in the PBST buffer at 37 °C, with the flow rate set to 25 µL/min and with lectin Concanavalin A (Con A, Sigma Aldrich) ranging from 1.6 to 12.8 µM. Each measurement consisted of three steps: washing with PBST (~15 min), lectin-glycan binding (30 min) and washing (30 min).

The kinetic parameters of the association (k_on_) and dissociation (k_off_) rate constants, as well as the dissociation (K_D_) constant, were determined using a simple ligand-receptor model as described earlier [39]. The viscoelastic index (VI) was calculated as a tangent of the slope angle obtained on the D(f) plots of each measurement (thanks to simultaneous measurements of changes in both parameters: frequency and dissipation factor; more details concerning this methodology can be found in [40]).

### 2.11. Lectin-ELISA Assay

The binding ability of the fluorescently-labeled lectin (Con A-FITC, Sigma Aldrich) to the glycans present on the cell surface was measured with a multi-plate reader with the procedure described in [39]. Cells were seeded into the 96-well plate at the density of 1 × 10^4^ cells/well. After 24 h of AEA treatment, cells were fixed with a 3.7% PFA solution and incubated with various concentrations of FITC-conjugated Con A (1.6–12.8 µM in PBST) for 30 min at RT and one concentration of the Hoechst 33,342 dye (1 μg/mL in PBS, Thermo Fisher Scientific) for 15 min at RT. Fluorescence was measured at the following wavelengths: 490_EX_/525_EM_ nm for Con A-FITC (glycans staining) and 350_EX_/461_EM_ nm for Hoechst (cell nuclei staining). The results for the Con A-FITC binding were first normalized by the cell nuclei number recorded for Hoechst staining and presented in the form of fluorescence intensity (in relative fluorescence units, RFU) dependence as a function of Con A–FITC concentration. The parameters of the received linear regression were used to determine the lectin binding ability.

### 2.12. Scratch Assay

Melanoma cell migration rate was analyzed using the scratch assay. Cells were seeded into the 24-well plate at the density of 3 × 10^5^ cells/well. After 24 h of AEA treatment, the scratch was made in the center of each well, the medium from each well was removed and replaced by a fresh medium (RMPI-1640 with 2% FBS) without the compound. Pictures were taken through the reverted optical microscope (CKX41, Olympus Corporation, Tokyo, Japan) in time points: 0 h (just after creating the scratch) and 24 h after the scratch was made. Next, by using the cellSens Dimension software on each picture, the crack was marked and the diameter value was registered. From each well the migration rate was calculated by dividing the scratch diameter obtained after 24 h by the scratch diameter at the starting point (0 h). The results were expressed as a percent of the complete overgrowth of the scratch by the cells.

### 2.13. Elasticity of Single Cells

The elasticity of a single cell treated with AEA was measured using a commercial atomic force microscope (XE120 model, Park Systems, Suwon, Korea) and an optical microscope to control the position of the gold-coated silicon nitride cantilevers (MLCT-C, Bruker Corporation, Billerica, Massachusetts, USA) with a nominal spring constant of 0.01 N/m. Cells were seeded at the density of 3 × 10^4^ cells/well on glass coverslips and next treated with 1 µM AEA or 0.07% *v*/*v* DMSO (CTR) for 24 h. All measurements were performed at RT in a medium without FBS.

Force curves were collected randomly from chosen cells from the region around the cell center. The force was set up to 4 nN, the approach velocity to 9 µm/s and a grid of 4 × 4 points on each cell was selected. The elastic modulus (E) values were calculated based on the subtraction of the two force curves: the calibration curve recorded on the glass coverslip without the cells and the other curves collected on a given cell [41]. The obtained force-versus-indentation-curve was analyzed by means of the Sneddon extension of the Hertz model assuming that the tip is an infinitely stiff indenter modeled by a parabola [42]. The results were presented for the indentation depth of 300 nm.

### 2.14. Actin Filaments and Cell Nuclei Visualization

The F-actin and nucleus of melanoma cells were stained as described previously [42]. Cells were seeded at the density of 3 × 10^4^ cells/well on glass coverslips and next treated with 1 µM AEA or 0.07% *v*/*v* DMSO for 24 h. The cells were washed with PBS, fixed with 3.7% PFA/PBS for 30 min at RT, washed with PBS, permeabilized with 0.1% Triton X-100 in PBS for 10 min at RT and again washed with PBS. Then, the actin filaments were stained with a phalloidin labeled with Alexa Fluor 488 (1:300, Life Technologies) in PBS for 30 min at RT and chromatin using the Hoechst 33342 dye (1 μg/mL) in PBS for 15 min at RT. Whole-cell images were collected with the excitation/emission 350 nm/461 nm for Hoechst 33342 and 495 nm/518 nm for phalloidin–Alexa Fluor 488 using the fluorescent microscope (IX71, Olympus).

### 2.15. Statistical Analysis

All experiments were performed in at least triplicate technical repetitions and in three biological repetitions. The data were presented as mean values ± standard deviations (SD) if possible. Statistical differences among the groups of data were analyzed by using a one-way ANOVA assay with the post hoc Dunnett’s test. The results of the *p*-value below 0.05 were considered significant.

## 3. Results

### 3.1. Melanoma Cell Viability after Anandamide Treatment

**Figure 1:**
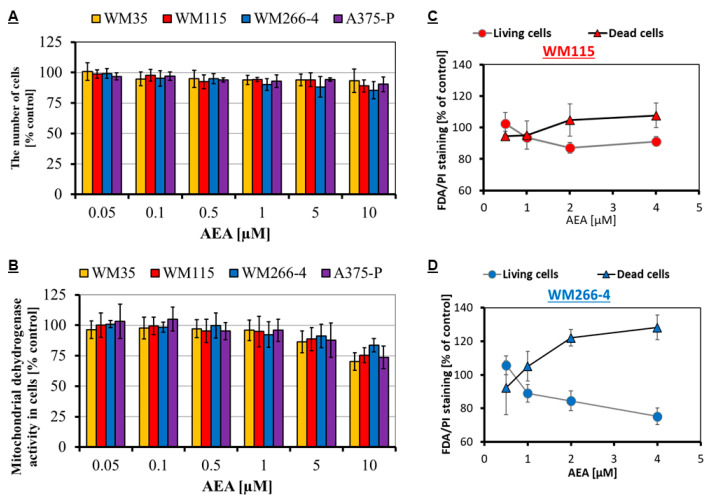
Influence of AEA on the number of melanoma cells calculated from CV assay (**A**), the mitochondrial dehydrogenase activity of melanoma cells checked by means of the MTT assay (**B**) and living/dead melanoma cell estimation based on the double FDA/PI staining for the primary VGP site WM115 cells—red (**C**) and metastatic WM266-4 cells—blue (**D**).

Three independent assays (MTT, CV and FDA/PI) were used to evaluate the influence of anandamide treatment on melanoma cell lines from various progression stages: RGP site (WM35), VGP site (WM115), metastasis to the lymph node (WM266-4) and from the solid tumor site (A375-P). After 24 h incubation of melanoma cells with 0.05–10 μM AEA, the number of melanoma cells stained with crystal violet was comparable to the control in the whole range of concentrations (~90% cells even for the highest AEA concentration; Figure 1A), but the mitochondrial dehydrogenase activity of melanoma cells was decreased by approximately 25% for 10 μM AEA (Figure 1B). The double FDA/PI staining of closely related melanoma cell lines (derived from the same patient) revealed that metastatic WM266-4 cells are more sensitive to the narrowed range 0.5–4 μM of AEA concentration than WM115 cells from the primary site (Figure 1C). With the increase in the AEA concentration, higher changes in the esterase activity in the living cells and the intercalation of propidium iodine to DNA in cells with damaged membrane were well visible. At the same time, the 1 μM AEA concentration seemed to only slightly affect the cells; thus, this concentration was selected for the following experiments.

### 3.2. Influence of AEA on Melanoma Cell Metabolism

**Figure 2:**
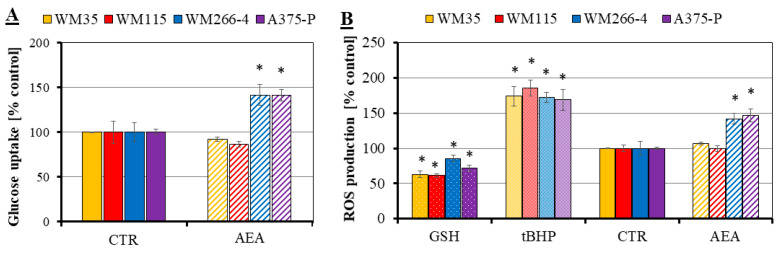
Influence of AEA on melanoma cell metabolism: measurements of the glucose uptake (**A**) and quantification of ROS production (**B**). The investigated cell lines are from the following stages of melanoma progression: RGP site (WM35—yellow), VGP site (WM115—red), metastasis to the lymph node (WM266-4—blue) and from the solid tumor site (A375-P—violet). Two additional controls were prepared: a positive control—cells treated with 100 μM tBHP and a negative control—cells treated with 5 mM GSH. The statistical significance of the *p*-value below 0.05 (*) was marked on the graph.

Changes in melanoma cell metabolism due to AEA treatment were investigated with two types of tests: the glucose uptake and the quantification of reactive oxygen species production by cells. A significant increase in the glucose uptake was observed only for metastatic cell lines incubated with 1 μM AEA concentration (~140% of control for both WM266-4 and A375-P cells; Figure 2A), a similar result was received for the quantification of ROS production by AEA-treated melanoma cells (140–150% of control for only metastatic WM266-4 and A375-P cells; Figure 2B). The results of the glucose uptake and quantification of ROS production by melanoma cells from the primary sites (WM35 and WM115) were not significantly influenced by anandamide treatment.

### 3.3. Changes in Glycosyltranferases Expression in the AEA-Treated Melanoma Cells

**Figure 3:**
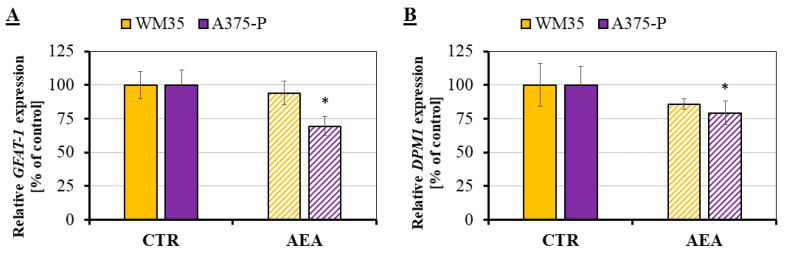
RT-qPCR analysis of *GFAT-1* (**A**) and *DPM1* (**B**) expression in AEA-treated melanoma cells. The investigated cell lines are from the following stages of melanoma progression: RGP site (WM35—yellow) and from the solid tumor site (A375-P—violet). The statistical significance of the *p*-value below 0.05 (*) was marked on the graph.

The mRNA expression of two glycosyltransferases (glutamine fructose-6-phosphate aminotransferase 1, GFAT-1; and dolichyl-phosphate mannosyltransferase subunit 1, DPM1) in melanoma cells incubated with 1 μM AEA were investigated by means of the RT-qPCR analysis (Figure 3A,B). Although the expression of both of these markers decreased for both primary WM35 cells and metastatic A375-P cells, a significant change was found only for the metastatic cells. The *GFAT-1* and *DPM1* expressions for metastatic cells were at the level of 69% and 79% of the control, respectively. On the other hand, WM35 expressed these markers at the level of 94% and 86% of control, respectively.

### 3.4. AEA-Induced Changes in L1-CAM Production by Melanoma Cells

**Figure 4:**
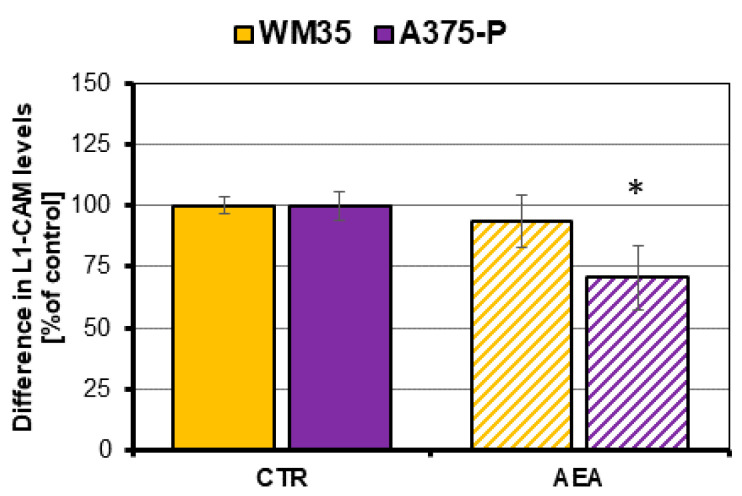
Difference in the L1-CAM glycoprotein production by AEA-treated melanoma cells. The investigated cell lines are from the following stages of melanoma progression: RGP site (WM35) and from the solid tumor site (A375-P). The statistical significance of the *p*-value below 0.05 (*) was marked on the graph.

With the in situ ELISA assay, the difference in the L1-CAM transmembrane glycoprotein with the N-linked high-mannose oligosaccharides production by melanoma cells was investigated after the cell treatment with 1 μM anandamide (Figure 4). The results showed that there were no differences in the protein level in the case of the melanoma cells from the primary RGP site (WM35). However, in association with the treated metastatic cells A375-P, a significant reduction in the L1-CAM protein amount was observed in comparison with the control (71% of control).

### 3.5. Biophysical Measurements of Changes in the Glycosylation Profile of AEA-Treated Melanoma Cells

**Figure 5:**
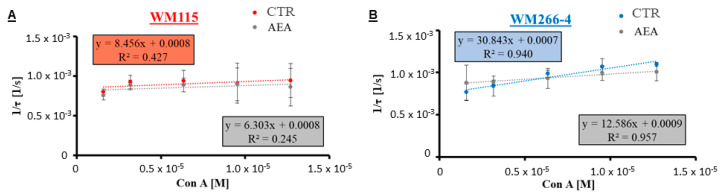
QCM-D results of the lectin–glycan binding measurements performed on AEA-treated and closely related (from the same patient) VGP cells (**A**) WM115 in red) and cells from the metastasis to the lymph node (**B**) WM266-4 in blue). The corresponding relations of the reverse in relaxation time plotted as a function of lectin Con A concentration with the established linear regressions were used to deliver the kinetic parameters of the lectin-glycan interaction (k_on_ and k_off_).

Changes in the glycosylation profile of melanoma cells induced by the 1 μM anandamide treatment were measured by means of the biophysical technique—the quartz crystal microbalance with dissipation monitoring. For these measurements, cells were cultured on gold sensors coated with polystyrene. Lectin Con A has a high specificity towards mannose and glucose residues, which are present in cell surface glycans. By using several concentrations of the lectin (1.6–12.8 μM) and measuring real-time changes in frequency while the lectin-glycan interaction takes place, the kinetic analysis of the binding was performed. The results of the simple ligand–receptor modeling are presented in Figure 5, where the reverse in relaxation time was plotted as a function of lectin concentration. From the linear regression, the association (k_on_) and dissociation (k_off_) rate constants, which describe the kinetics of the lectin–glycan complex formation, were determined (full data available in the Supplementary Materials Table S1). Finally, from the dependency between the association and dissociation rate constants, the lectin affinity towards cell surface glycans (dissociation constant, K_D_) was calculated (Table 1). Lectin affinity was significantly higher towards metastatic melanoma cells (WM266-4 and A375-P-controls; lower K_D_ values = 0.23 and 0.36 nM, respectively) than for the cells from the primary tumor sites (WM35 and WM115-controls; higher K_D_ values = 1.80 and 0.95 nM, respectively). However, after the AEA treatment of cells, the lectin affinity towards the metastatic cells decreased significantly (3.1x and 4.6x higher K_D_ values for WM266-4 and A375-P, respectively); whereas for the primary tumor site cells, it changed only slightly (1.8x and 1.5x higher K_D_ values for WM35 and WM115, respectively) (Table 1).

**Figure 6:**
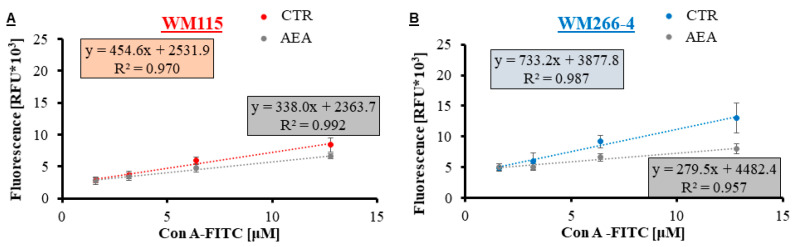
The results of the lectin-ELISA assay for AEA-treated and closely related (from the same patient) melanoma VGP cells ((**A**) WM115 in red) and cells from the metastasis to the lymph node ((**B**) WM266-4 in blue), where the fluorescent intensity was plotted as a function of the bound lectin Con A-FITC to the glycans present on the investigated cells.

With the lectin-ELISA assay, the affinity results of the lectin–glycan interaction performed on melanoma cells treated with 1 μM AEA were verified. In this assay, similar Con A concentrations were used (1.6–12.8 μM) similar to those used for the QCM-D experiments, but the utilized lectin had to be labeled fluorescently (e.g., Con A-FITC). The results of the measured fluorescence were plotted against the concentration of lectin (Figure 6) and the received slope and intercept values provided information on the changing lectin–glycan interaction (full data available in the Supplementary Materials Table S2). The relation between the obtained parameters of the linear regression enabled the comparison of the binding ability of Con A to glycans (Table 2). Con A binding ability (b/a relation) of the control cells decreased with the progression stages of melanoma from 20.7 for primary WM35 cells to 4.5 for metastatic A375-P cells. Treatment of melanoma cells with anandamide from both primary sites (RGP and VGP) did not change the binding ability (a similar value of b/a ratio, 1.0–1.3); however, in the case of both metastatic cell lines, the binding ability decreased significantly (a high value of b/a ratio, 3.0–4.1). These b/a ratio values were comparable to the earlier obtained K_D_ ratio values.

**Figure 7:**
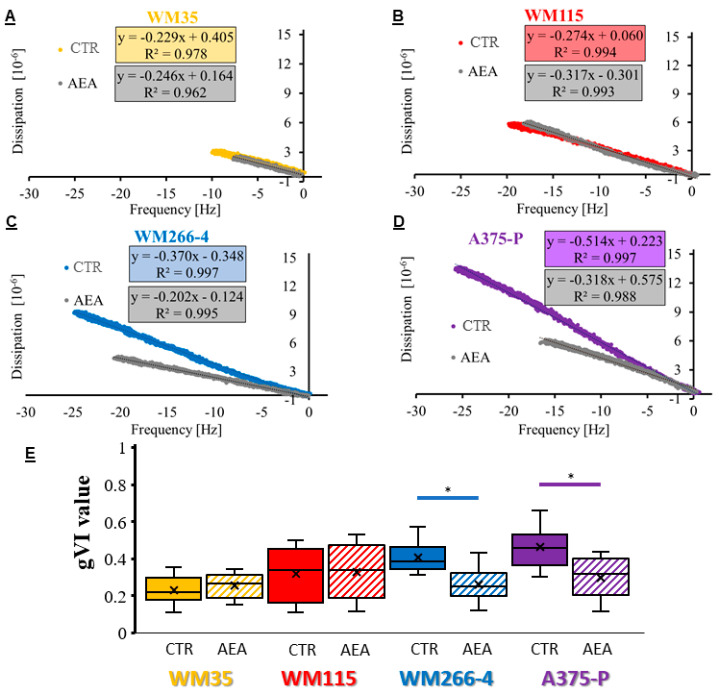
Df plots received for the 6.4 μM Con A concentration of the lectin-glycan interaction performed on AEA-treated melanoma RGP cells (**A**)—WM35), VGP cells (**B**)—WM115), metastasis to the lymph node (**C**)—WM266-4) and solid tumor metastatic cells (**D**)—A375-P). (**E**)—Overview of the obtained viscoelastic index values for Con A concentrations (1.6–12.8 μM) interacting with glycans of melanoma cells treated with AEA. The statistical significance of the *p*-value below 0.05 (*) was marked on the graph.

During the QCM-D experiments, simultaneously to the frequency measurements, the dissipation parameter was registered. Plotting raw data of frequency vs. dissipation (Df plot) enabled the analysis of the glycan Viscoelastic Index (gVI) value equal to the tangent of the angle made by the estimated linear regression (Figure 7). This prognostic factor shows the differences in the viscoelastic properties of the created lectin–glycan complexes. The glycan viscoelastic index values of the non-treated cells increased with the progression stages of melanoma. AEA treatment of both types of the melanoma cells from the primary site (RGP-WM35 and VGP-WM115) slightly increased the gVI values (1.1 and 1.2, respectively), whereas metastatic melanoma WM226-4 and A375-P cells presented significantly decreased the gVI values (Table 3).

### 3.6. Anandamide-Induced Changes to Melanoma Cell Migration

**Figure 8:**
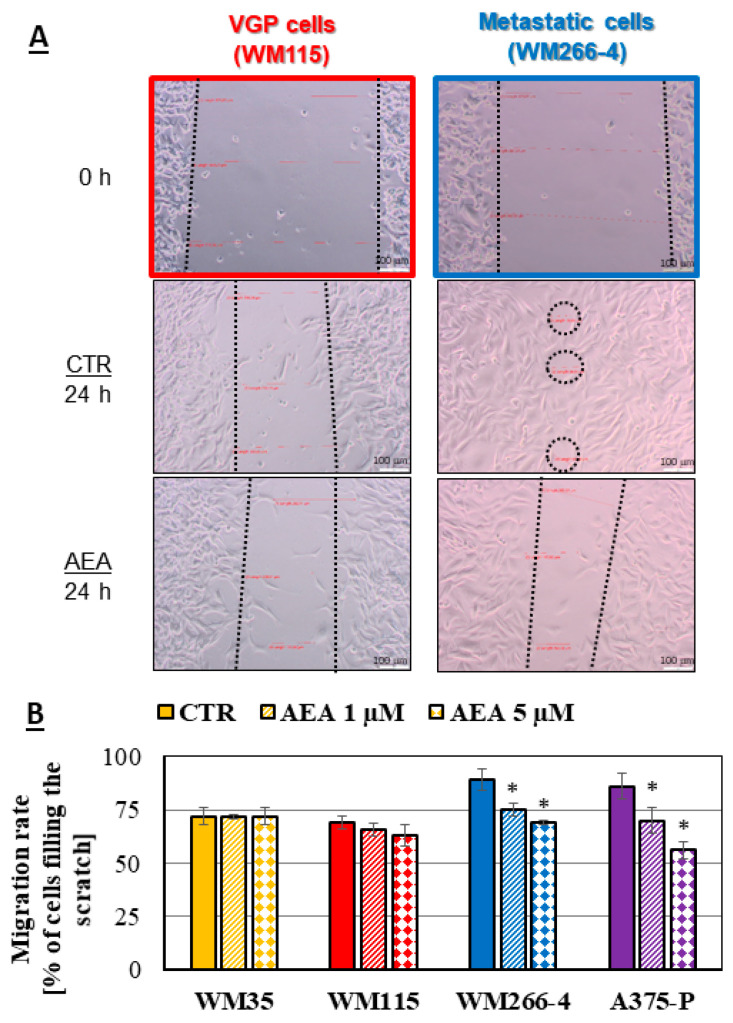
Migration rate analysis of melanoma cells: (**A**)—the representative pictures of the closely related WM115 and WM266-4 melanoma cells (from the same patient) after creating the scratch (0 h) and 24 h later after the 1 μM AEA treatment (scale bar—100 μm). (**B**)—the calculated migration rate of different melanoma cells after 1 μM and 5 μM AEA treatment. The statistical significance of the *p*-value below 0.05 (*) was marked on the graph.

The difference in the cell migration rate was investigated with the use of the scratch assay (Figure 8A,B). Exceptionally in this analysis, two anandamide concentrations (1 and 5 μM) were investigated. The migration rate of the primary cells from the RGP site did not change after the AEA treatment (approximately 1% of change), but at the same time for the cells from the VGP site, it was reduced by 3% for 1 μM AEA and 6% for 5 μM AEA in comparison with the untreated cells. In the case of both metastatic cell lines the migration rate was reduced by 14–16% for 1 μM AEA and 20–30% for 5 μM AEA (Figure 8B).

### 3.7. Unchanged Elastic Properties of Melanoma Cells Treated with AEA

**Figure 9:**
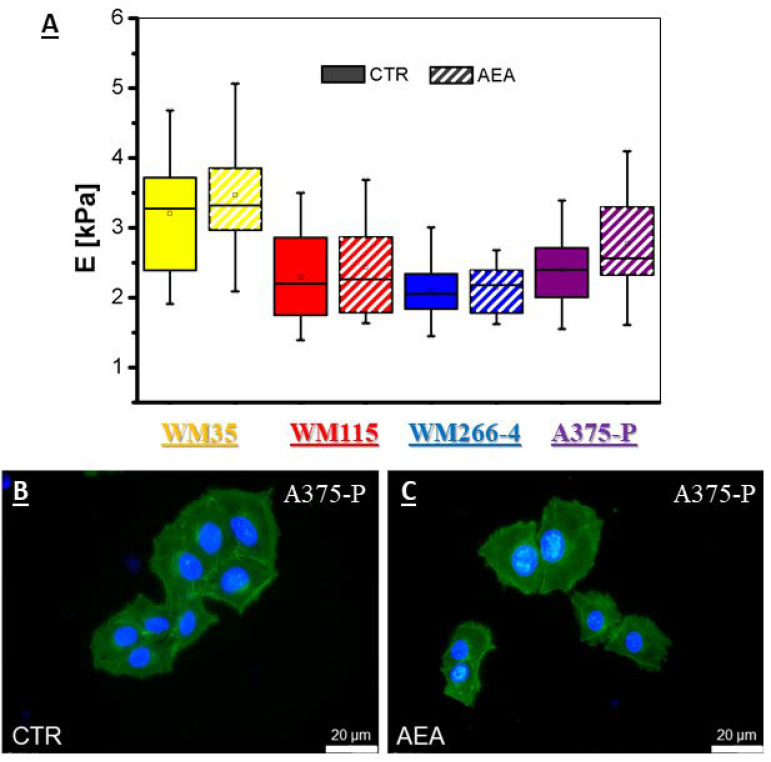
(**A**)—the calculated elastic modulus of different melanoma cell lines treated with 1 μM AEA at the indentation depth of 300 nm. Cell lines: WM35—primary RGP site; WM115—primary VGP site; WM266-4—metastasis to the lymph node; A375-P—solid tumor metastatic site. The representative pictures of A375-P melanoma cells: control (**B**) and treated with 1 μM AEA (**C**) after cell staining with phalloidin labeled with Alexa Fluor 488 (ex. 495 nm, em. 518 nm) and Hoechst 33342 (ex. 350 nm, em. 461 nm).

To check the influence of AEA on the mechanical properties of melanoma cells, two types of analyses were performed: measurements of cell elasticity with the atomic force microscope and visualization of the cytoskeleton by the F-actin staining. Incubation of melanoma cells with AEA in general caused only minimal changes in the mechanical properties of cells (by approximately 14% of value for metastatic A375-P cells; Figure 9A, Table 4), and it did not cause visible changes in cell morphology in terms of potential actin filaments reorganization (Figure 9B,C).

## 4. Discussion

In this study, we checked the influence of anandamide, an endogenous cannabinoid, on melanoma cell properties related with metabolism, glycosylation and migration. Four different commercial melanoma cell lines were used as a basic research model, which were derived from the following stages of tumor progression and thus represent the radial growth phase (WM35), the vertical growth phase (WM115), the lymph node metastasis (WM266-4) and the solid tumor metastasis (A375-P). It is worth mentioning that the WM115 and WM266-4 cells were obtained from the same patient; thus, they constituted a reliable in vitro research model. Although commercial cell lines are just one of the possible models for studying the potential anti-cancer activity of compounds (among the ex vivo patient-derived models and in vivo rodent models, which better reflect the heterogeneity of various tumors [43,44]) basic research on tumors needs to be performed first on models that are widely recognized (as for the results to be comparable in different laboratories), before the more advanced models can be applied. However, as in any other model, some limitations are also related to the usage of commercial cell lines as models. In general, the established melanoma cell lines are said to maintain their properties in a stable manner indicated by chromosomal analyses of these cells at the time of isolation, after 12 passages in culture and after prolonged (more than 6 months) culture [45]. Furthermore, the artificial environment and laboratory manipulations, for sure, can be stressful conditions for cells during which increased numbers of mutations in the genomic DNA can arise. Moreover, in some cases the continual passage of the cell line is accompanied by an extensive clonal selection and in consequence a loss of heterogeneity [45,46]. That is why we conventionally use melanoma cell lines only up to the 20th passage for research. Moreover, an important limitation of single cell line culture in both 2D/3D is the lack of the immune components, which decreases this model’s value in tumor immunotherapy. Yet, in some studies to improve the model, researchers have tried to establish a coculture of immune cells with cell lines and organoids [47].

The first experiments in this basic research, performed on commercially derived melanoma cells, were dedicated to select only these concentrations of AEA, which would not significantly reduce the number of living cells in the samples. The CV assay was performed to measure the number of cells, the MTT assay to present the cells in which the enzyme mitochondrial dehydrogenase is still active and the FDA/PI assay to simultaneously quantify cells with active enzyme esterase (living cells) and with damaged cell membrane (dead cells). The results of all three tests were consistent and show that concentrations higher than 5 μM can have a negative impact on melanoma cells. Metastatic cells were slightly more sensitive to the increasing AEA concentrations as revealed with the double staining assay. These results are in agreement with the research presented by Adinolfi et al. [33], where the IC_50_ mean value for the A375 melanoma cell line was approximately 13.5 ± 0.3 μM. Gomez et al. [48] observed a significant decrease in the mitochondrial dehydrogenase activity of chondrocytes after treatment with a 15 μM AEA concentration (down to 40% of active cells) and with 1–5 μM concentrations low influence with the decreasing values of 90% to 75% of active cells, respectively. The usage of a neuronal cell model (the PC-12 cell line) by Sarker et al. [49] exhibited a similar influence of AEA on these cells similar to the others. Concentrations lower than 7.5 μM did not reduce significantly the mitochondrial dehydrogenase activity in the cells (80% of active cells), but higher concentrations such as 10 μM were toxic to the cells (approximately 30% of active cells). All these results were consistent in showing that the AEA concentrations close to 10 μM may show toxicity to different kinds of cells including melanoma. However, interesting results were also obtained by Bilmin et al. [50], who studied the influence of AEA on glioma cells cultured in medium with and without fetal bovine serum for 24 h. FBS in the cell culture, provides many essential nutrients and growth factors that facilitate cell survival and proliferation. When glioma cells were cultured in a full medium (with 10% FBS) and several AEA concentrations (up to 30 μM) were tested, the mitochondrial dehydrogenase activity of the cell was stimulated (120% of active cells for 7.5 μM AEA) or had a rather low impact (80% of active cells for 30 μM). In a medium without FBS, all studied concentrations presented a significantly low mitochondrial dehydrogenase activity of the cells ranging from 60% for 3.75 μM AEA, 50% for 7.5 μM and even down to 25% of active cells for the 30 μM AEA concentration. These results suggest that the addition of a 10% FBS somehow prevents the cells from the anandamide treatment. These results were partially explained by De Petrocellis et al. [51]. In the presence of a bovine serum albumin the potency of anandamide at a TRPV1 receptor is markedly reduced, which suggests that BSA can prevent the uptake of anandamide by first interfering with the carrier-mediated internalization of this compound and next the subsequent activation of the receptor. In our case, the experiments were carried out in the presence of a FBS concentration of 10% *v*/*v*. In order to maintain the current conditions, the FBS addition in the growth medium was continued.

Most cancer therapies are focused on the application of inhibitors of the MAPK pathway or on targeting metabolic pathways [52]. Anandamide has a confirmed influence on some of these pathways, for example, the extracellular regulated kinase 1/2 (ERK1/2), the p38 mitogen-activated protein kinase (p38 MAPK), the nuclear factor kappaB (NF-κB) and the c-jun N-terminal kinase (JNK) were activated by the short-time incubation of 10 μM AEA with human skeletal muscle cells. Interestingly, 10 μM AEA significantly increased the glucose uptake in these cells (300% of control), which was not accompanied by the increase in the glucose transporters (including GLUT1 or GLUT4) abundance [53]. Thus, we decided to check if anandamide had the same influence on the glucose uptake performed by melanoma cells and what effect it had on the results from the intensive metabolism production of reactive oxygen species, as the disturbance in the glucose uptake is typically followed by changes in the natural by-products of the metabolism in the form of various reactive species (oxygen, ROS; or nitrogen, RNS) [25]. With both performed analyses we received a significant increase in the resulting values of glucose uptake and ROS production (approximately 150% of control) for only the metastatic cell lines treated with 1 μM AEA. Park et al. [54] suggested that a significant rise of ROS levels (depending on the cell line 200–350% of control) caused by the 18 h treatment with AEA, in general, plays a partial role in the anticancer effect of this endocannabinoid on the head and neck squamous cell carcinoma (HNSCC) lines. Moreover, a 2 h incubation with 10 μM AEA induced a significant increase (30-times) in the intracellular ROS accumulation in cholangiocytes isolated from BDL mice [55]. These results are consistent with ours in terms of the AEA-sensitive metastatic melanoma cells and enable presuming that the disturbance in the glucose uptake further results in the changed cell metabolism and stimulation of ROS production.

Due to the existing connection between glycolysis (the metabolic pathway that converts extracellular glucose into pyruvate) and its side path—the Hexosamine Biosynthetic Pathway (HBP), one can expect that AEA-induced changes in the glucose uptake process in melanoma cells may also affect the cell glycosylation profile. HBP is the first but crucial part of the complex glycan biosynthesis pathway, which takes place in all organisms, from which the product UDP-GlcNAc is a major substrate for most kinds of glycosylations excluding glycosphingolipids [56,57,58]. Changes occurring in the N- or O-glycans can be firstly investigated by checking the expression of genes encoding enzymes related to the glycan biosynthesis pathway, such as glutamine fructose-6-phosphate aminotransferase (GFAT) or dolichyl-phosphate mannosyltransferase (DPMs). The GFAT family mainly consists of two isoforms GFAT-1 and GFAT-2, which are the rate-limiting enzymes of the HBP pathway, because they convert fructose 6-phosphate to glucosamine 6-phosphate [59]. DPMs enzyme is built from the three subunits (one catalytic subunit DPM1, and two regulatory subunits DPM2 and DPM3) and it is responsible for generating dolichol phosphate-mannose (Dol-P-Man) from GDP-mannose (GDP-Man), and dolichol phosphate (Dol-P) [60,61]. Thus, these two enzymes are essential for the first few steps of the glycan biosynthesis and the difference in their expression may result in a varied degree of cell glycosylation, as well as the possible switch in the glucose metabolism in cells. We observed a significant reduction in both *GFAT-1* and *DPM1* expressions in metastatic melanoma cells treated with anandamide, which may suggest that the biosynthesis of glycans in these cells was downregulated. As shown in the literature, *GFAT-1* and *DPM1* overexpression is of a cell/patient specific manner [62], but the activity of the enzymes encoded by the investigated genes may also be influenced by several genetic and metabolic alterations [63]. During the epithelial-to-mesenchymal transition (EMT) of A549 lung cancer cells, the glucose uptake was intensified, but instead of increasing the glycolysis, the glucose was directed to the HBP pathway [64]. *GFAT* is known to be overexpressed in many cancer types that present EMT features and the decrease in the *GFAT* expression may inhibit the EMT [65]. The *GFAT* upregulation also promotes cell migration and its expression correlates with poor clinical outcomes [66]. Melanoma cells undergo the epithelial-to mesenchymal-like transformation [36]; thus, for the tested cell lines in this research, it is understandable that the base level of the *GFAT-1* expression for melanoma cells was significantly higher for metastatic A375-P cells than for the primary WM35 cells (approximately 138%, data available in the Supplementary Materials Table S1). At the same time, the 24 h incubation of A375-P metastatic cells with anandamide resulted in the decreased values of the relative *GFAT-1* expression indicating possible inhibition of the EMT process. Altered DPMs gene expression level and enzyme activity (the only known mannose donor for mannosylation in the lumen of the endoplasmic reticulum) in humans is linked to cancer and congenital disorders of glycosylation [67,68]. The *DPM1* gene plays a crucial role in colorectal cancer [62] and breast cancer, but its expression measured by means of the microarray analysis in tissue samples obtained from patients with breast cancer showed significant differences in values ranging from 761 up to even 5424 [69]. Considering this information, the obtained result confirmed the cell-dependent differences in the overexpression of the investigated genes. However, only the study of the enzyme activity could show if the observed difference in these gene expression levels really influenced the glycosylation of cells. In some cases, the upregulation of genes does not guarantee the increased activity of the encoded enzymes, which may be the case of the A375-P cells’ lower result than for the WM35 cells in terms of the basal level of the *DPM1* gene expression (approximately 62%, data available in the Supplementary Materials Table S1).

On the other hand, the abnormal oligosaccharides are widely expressed on the cell adhesion molecules (CAMs) such as β1 and β3 integrin subunits, a few of the α integrin subunits, CD44 as well as cadherins. The L1 cell adhesion molecule (L1-CAM, CD171) is a 200–220 kDa transmembrane glycoprotein with the N-linked high-mannose oligosaccharides. Its expression was identified in primary and cutaneous metastases of melanoma, but not in melanocytic nevi and melanocytes. The presence of L1-CAM in carcinomas increases the spreading of tumor cells by enabling cell migration, invasion and it promotes the EMT. Therefore, quantification of L1-CAM glycoprotein in cells may be considered as a marker of prognosis in several types of cancer including melanoma (high protein level—low chances for survival) and its occurrence accompanies a migratory phenotype of cells [70,71,72]. The obtained results of the decrease in this protein amounts in metastatic A375-P cells treated with a 1 μM concentration of anandamide in comparison with the control cells, confirm that the aggressive phenotype of these cells was temporarily blocked, whereas AEA had only a slight influence on the L1-CAM protein in the primary WM35 cells.

To biophysically analyze changes in the glycosylation profile of cells, we used the QCM-D technique and measured the lectin Con A-glycan binding on melanoma cells treated with anandamide in real-time. The QCM-D is a highly specific biophysical method, which allows the investigated interaction process occurring between two molecules (one bound to the surface of the sensor and the second one delivered in a flow rate) to take place with the maximum mass sensitivity of 17.7 ng/(cm^2^∙Hz) [73]. Lectin concanavalin A, used for this study, binds to mannose and glucose residues of surface glycans such as L1-CAM (transmembrane protein with N-linked high-mannose oligosaccharides) [74]. At the same time, melanoma cells were seeded onto the QCM-D sensors. The Con A affinity towards metastatic cells was significantly reduced after anandamide treatment (higher K_D_ values) at the same time the glycan viscoelastic index was significantly reduced (lower gVI values). This suggests a major difference in the glycosylation profile of metastatic melanoma cells treated with AEA. Although this research is the first to show the anandamide influence on cells by means of the QCM-D method, much is already known in terms of the glycosylation profile of various melanoma cells, including cell lines and primary cells isolated from a patient with confirmed melanoma metastasis [36,39,40]. With the tumor progression, the protein glycosylation on cells changes from short and lowly-branched surface glycans (such as on the WM35 cells of the primary RGP site) to long and highly branched oligosaccharides (present on the A375-P metastatic cells), thus the lectin Con A affinity for metastatic cells is higher than for the primary cells and the glycan viscoelastic index significantly increases. Because melanoma tumors are highly heterogenic, melanoma cells isolated from the same patient often differ in metastatic potential, for example, established melanoma subcultures presented a varied doubling time [75], as well as disparate cell elasticity (AFM analysis) and glycosylation profile (QCM-D analysis; [36]). However, all results were consistent: MM7 and MM9 populations were the quickest to divide (low value of doubling time), less resistant to deformation (low E values) and with high metastatic potential (low K_D_ value and high gVI value). Taking this all into consideration, the results for the AEA treatment of melanoma cells clearly show a significant change in the metastatic potential of WM266-4 and A375-P cells to be of less aggressive phenotype.

Cell motility depends strongly on the N-linked glycosylation and changes which occur during the altered glycosylation [76]. For example, the upregulation of *GFAT* genes promotes cell migration and their expression correlates with poor clinical outcomes [66]. This process is mainly controlled by the cadherin switch, which facilities the migration of cells from the surrounding tissue to other organs and promotes the process of angiogenesis [77,78]. Moreover, cell migration is promoted by the integrin-specific signaling pathways that is responsible for changes in the focal adhesion [76]. The migration can also be correlated with the changing amount of surface glycoproteins, such as L1-CAM. Its reduction may appear as a diminished cell migration [70,71] and the tumor cells could be eliminated from the organism by, for example, tissue excision. Due to the observed changes in the interaction of lectin with glycans on the investigated melanoma cells caused by treatment with the selected concentration of anandamide, the modification of the migration rate of these cells was also possible and needed to be checked experimentally. As our results show, metastatic cell migration was significantly reduced by 1 μM and 5 μM AEA treatment. The difference in cell motility was not induced by the changes in the cell proliferation rate, as anandamide does not change this property of melanoma cells (the results of BrdU assay are available in the Supplementary Materials Figure S2). Similarly, when a highly invasive human breast cancer cell line (MDA-MB-231) was treated with a 10 μM concentration of the metabolically stable anandamide analogue 2-methyl-2′-F-anandamide (Met-F-AEA), the inhibition of the adhesion as well as migration of cells occurred (a change of approximately 20–30%). This was mainly due to the modulation of the kinase FAK phosphorylation (a twice lower amount of the pFAK kinase in the treated cells) [79]. Furthermore, Joseph et al. [80] suggested that the inhibitory function of anandamide on the migration of tumor cells was mediated by the CB1 receptor. These results enable crating a hypothesis that the CB1 receptor activation might represent a novel therapeutic strategy to slow down the growth of several carcinomas and to inhibit their metastatic diffusion in vivo. This hypothesis has been confirmed recently by Carpi et al. [81], who proved that the CB1 receptor might function as a tumor-promoting signal in the human cutaneous melanoma by means of the real-time PCR and Western blot analysis. After all, AEA is a well-known agonist ligand for both types of the endocannabinoid receptors with a higher affinity for the CB1 receptor than CB2 [82].

At some point, the decrease/increase in cell migration can be influenced by changes in cell elastic properties, which can be measured by atomic force microscope or observed by cell staining of the cytoskeleton proteins such as actin filaments. Hohmann et al. [83] studied the influence of a 10 μM anandamide derivative (chloroanandamid, ACEA) on the mechanical properties of 2 different glioblastoma cell lines and its influence did not exceed 10% difference from the control cells. However, the results presented some differences for each cell line: the highly proliferating U87 cells had decreased elastic modulus value, whereas for U138 cells with a slower proliferation the increase in E value was registered. In case of our results no significant influence was observed on the E values and reorganization of F-actin in primary as well as metastatic melanoma cells treated with anandamide confirming that changes in the cell migration were not influenced by the mechanical properties of cells.

## 5. Conclusions

Summarizing all the obtained results, the commercially derived primary RGP and VGP melanoma cells in general were resistant to AEA treatment (no significant changes in the studied parameters were noted). On the other hand, the influence of the 1 μM anandamide on the commercially derived metastatic melanoma cells was significant for the following analyzed properties: glucose uptake (increase by 40%), ROS production (increase by 40–50%), the expression of genes encoding glycosyltransferases (decrease for *GFAT-1* by 31% and for *DPM1* by 21%), the amount of L1 cell adhesion molecule in cells (decrease by 29%), lectin affinity toward cell surface glycans (3.1–4.6-times lower), glycan viscoelastic index (2-times lower) and the cell migration rate (reduced by 14–16%). At the same time, changes in migration rate were not influenced by the mechanical properties of cells, which were similar for the AEA-treated and control cells. Thus, AEA changes the metabolism, glycosylation profile and migration of metastatic melanoma cells, and should be recommended for a future check of its potential usage in the combinatory therapy for advanced melanoma stages on more advanced models.

## Figures and Tables

**Table 1:** Results of the QCM-D kinetic analysis of the studied lectin–glycan interaction performed on melanoma cells. K_D_ is the dissociation constant/lectin affinity towards cell surface glycans. The K_D_ ratio was calculated as the AEA/DMSO relation.

Cell Type/Cell Line	Sample	K_D_ [nM]	K_D_ Ratio
RGP site—WM35	CTR	1.80	1.8
	AEA	3.15	
VGP site—WM115	CTR	0.95	1.5
	AEA	1.43	
Metastasis to the lymph node—WM266-4	CTR	0.23	3.1
	AEA	0.72	
Solid tumor metastasis—A375-P	CTR	0.36	4.6
	AEA	1.66	

**Table 2:** Results of the lectin-ELISA analysis of the studied lectin–glycan interaction performed on AEA-treated melanoma cells. The b/a relation was calculated by using the parameters of the linear regression, where a is the slope and b the intercept. The b/a ratio was calculated as the AEA/DMSO relation.

Cell Type/Cell Line	Sample	b/a Relation	b/a Ratio
RGP site—WM35	CTR	20.7	1.0
	AEA	21.1	
VGP site—WM115	CTR	5.6	1.3
	AEA	7.0	
Metastasis to the lymph node—WM266-4	CTR	5.3	3.0
	AEA	16.0	
Solid tumor metastasis—A375-P	CTR	4.5	4.1
	AEA	18.6	

**Table 3:** Results of the glycan viscoelastic index analysis of the AEA-treated melanoma cells received from the QCM-D measurements. The gVI value equal to the tangent of the angle made by the estimated linear regression for the data present on the Df plots. The gVI ratio was calculated as the AEA/DMSO relation.

Cell Type/Cell Line	Sample	gVI	gVI Ratio
RGP site—WM35	CTR	0.229	1.1
	AEA	0.246	
VGP site—WM115	CTR	0.274	1.2
	AEA	0.317	
Metastasis to the lymph node—WM266-4	CTR	0.370	0.5
	AEA	0.202	
Solid tumor metastasis—A375-P	CTR	0.514	0.6
	AEA	0.318	

**Table 4:** Results of the elastic modulus (E) analysis of the AEA-treated melanoma cells received from the AFM measurements.

Cell Type/Cell Line	Sample	E [kPa]	E Ratio
RGP site—WM35	CTR	3.22 ± 0.86	1.08
	AEA	3.47 ± 0.80	
VGP site—WM115	CTR	2.29 ± 0.64	1.06
	AEA	2.44 ± 0.64	
Metastasis to the lymph node—WM266-4	CTR	2.14 ± 0.39	1.00
	AEA	2.14 ± 0.32	
Solid tumor metastasis—A375-P	CTR	2.45 ± 0.49	1.14
	AEA	2.80 ± 0.65	

## Data Availability

The data presented in this study are available on request from the corresponding authors.

## Supplementary Materials

The following supporting information can be downloaded at: https://www.mdpi.com/article/10.3390/cancers14061419/s1, Figure S1: RT-qPCR analysis of *GFAT-**1* and *DPM1* expression in melanoma cells from the RGP site (WM35—yellow) and from the solid tumor site (A375-P—violet); Figure S2: Proliferation rate analysis of melanoma cells after 24 h treatment with 1 μM and 5 μM anandamide, followed by a 2 h incubation with 10 μM BrdU. Then, melanoma cell proliferation rate was evaluated with the use of the BrdU Cell Proliferation Assay Kit (Merck) according to the producers protocol. Cell lines: WM35 (RGP site), WM115 (VGP site), WM266-4 (lymph node metastasis), A375-P (solid tumor metastasis); Table S1: The full results of the QCM-D kinetic analysis of the studied lectin-glycan interaction performed on melanoma cells; Table S2: The full results of the lectin-ELISA analysis of the studied lectin-glycan interaction performed on AEA-treated melanoma cells.
