# Supplementary material for: Anandamide-Modulated Changes in Metabolism, Glycosylation Profile and Migration of Metastatic Melanoma Cells
Source: Cancers (Basel). 2022 Mar 10;14(6):1419. doi: 10.3390/cancers14061419 (PMC8946642; doi:10.3390/cancers14061419)
Supplement: Supplementary file 1 [file cancers-14-01419-s001.zip › cancers-1608567-SI.pdf]

cancers

MDPI

# Anandamide-modulated changes in metabolism, glycosylation profile and migration of metastatic melanoma cells

**Anna Sobiepanek, Małgorzata Milner-Krawczyk, Paulina Musolf, Tomasz Starecki and Tomasz Kobiela**

## Supplementary materials

**Table S1.** The full results of the QCM-D kinetic analysis of the studied lectin-glycan interaction performed on melanoma cells. Legend: $k_{on}$ – the association rate constant, $k_{off}$ – the dissociation rate constants, $K_D$ – the dissociation constant / lectin affinity towards cell surface glycans. The $K_D$ ratio was calculated as the AEA/DMSO relation.

| Cell Type/Cell Line | Sample | $k_{on}$ [1/M*s] | $k_{off}$ [1/s] | $K_D$ [nM] | $K_D$ Ratio |
|---|---|---|---|---|---|
| RGP site – WM35 | CTR | 5.010 | 0.0009 | 1.80 | 1.8 |
| | AEA | 2.860 | 0.0009 | 3.15 | |
| VGP site – WM115 | CTR | 8.456 | 0.0008 | 0.95 | 1.5 |
| | AEA | 6.303 | 0.0008 | 1.43 | |
| Metastasis to the lymph node – WM266-4 | CTR | 30.843 | 0.0007 | 0.23 | 3.1 |
| | AEA | 12.586 | 0.0009 | 0.72 | |
| Solid tumor metastasis – A375-P | CTR | 19.627 | 0.0007 | 0.36 | 4.6 |
| | AEA | 4.810 | 0.0008 | 1.66 | |

**Table S2.** The full results of the lectin-ELISA analysis of the studied lectin-glycan interaction performed on AEA-treated melanoma cells. The b/a relation was calculated by using the parameters of the linear regression, where a is the slope and b the intercept. The b/a ratio was calculated as the AEA/DMSO relation.

| Cell Type/Cell Line | Sample | a Values | b Values | b/a Relation | b/a Ratio |
|---|---|---|---|---|---|
| RGP site – WM35 | CTR | 433.2 | 8987.2 | 20.7 | 1.0 |
| | AEA | 407.7 | 8588.7 | 21.1 | |
| VGP site – WM115 | CTR | 454.6 | 2531.9 | 5.6 | 1.3 |
| | AEA | 338 | 2363.7 | 7.0 | |
| Metastasis to the lymph node – WM266-4 | CTR | 733.2 | 3877.8 | 5.3 | 3.0 |
| | AEA | 279.5 | 4482.4 | 16.0 | |
| Solid tumor metastasis – A375-P | CTR | 1113.4 | 5011.9 | 4.5 | 4.1 |
| | AEA | 317.6 | 5897.4 | 18.6 | |

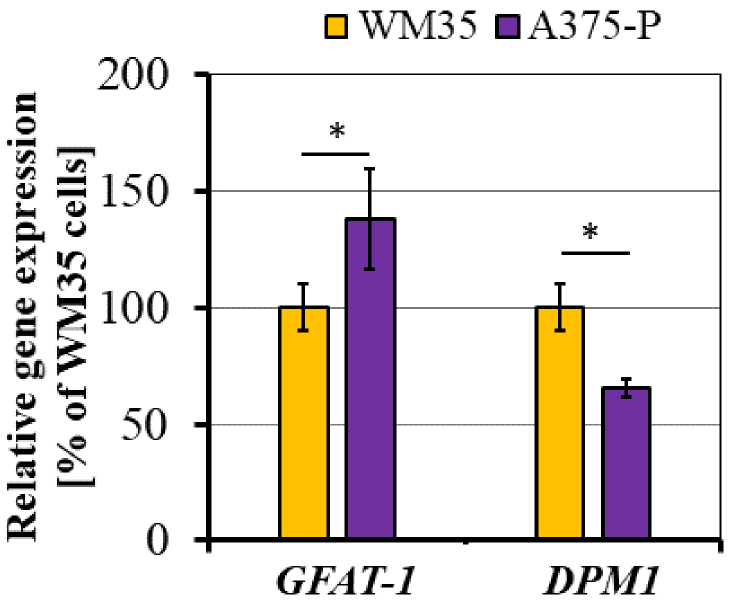


**Figure S1.** RT-qPCR analysis of *GFAT-1* and *DPM1* expression in melanoma cells from the RGP site (WM35 - yellow) and from the solid tumor site (A375-P - violet). The statistical significance of the p-value below 0.05 (*) was marked on the graph.

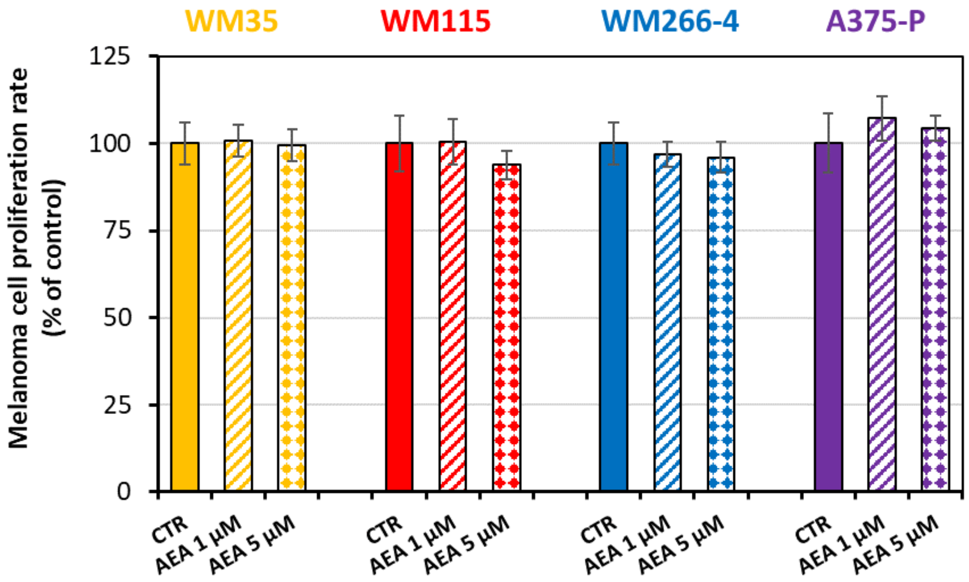


**Figure S2.** Proliferation rate analysis of melanoma cells after 24 hour treatment with 1 μM and 5 μM anandamide, followed by a 2 hour incubation with 10 μM BrdU. Then, melanoma cell proliferation rate was evaluated with the use of the BrdU Cell Proliferation Assay Kit (Merck) according to the producers protocol. Cell lines: WM35 (RGP site), WM115 (VGP site), WM266-4 (lymph node metastasis), A375-P (solid tumor metastasis).
